# A Concise Treatise on Operative Surgery

**Published:** 1838-04

**Authors:** 


Art. II.—*A concise Treatise on Operative Surgery; describing the Methods adopted by the English, Continental, and American Surgeons: selected for the use of junior Practitioners and Students. Illustrated by twelve Plates.* By W. P. Cocks, Surgeon.—*London,* 1837. 8vo. pp. 375.

Mr. Cocks's work is confessedly a selection. The plan of it is to give an account of the mode of performing operations, without any mention of the symptoms or circumstances which render such operations necessary. To this plan we should have very decided objections, even if it were ably and skilfully effected. To detail the mere manual part of surgery,—to string together receipts for the mode of doing operations, without first giving the student and young practitioner, for whom the work is "selected," some guide as to when he ought to operate, and when he ought not, appears to us extremely injudicious. The omission, too, of the slightest hint as to the probable failure or success of the different operations is very objectionable. The plan of this work, then, we think, is decidedly bad; and we are sorry that a sense of justice to our readers compels us to add, that the manner in which it is executed is slight, superficial, careless, and not unfrequently erroneous. Mr. Cocks states in the preface, that "the operator, referring to the index, puts his finger at once upon the best recorded method of accomplishing his design; and, with the text before him, he can act promptly and efficiently." We refrain from formally ridiculing the idea of Mr. Cocks's work being placed upon a reading-desk, or in the hands of an assistant, in order that the operator's knife should follow the direction of the text; but we must observe, that "the finger" can *not* "be put at once" upon the "best recorded method;" for the simplest of all possible reasons, because the selector first describes one way of operating; and then "another method," and "another method," and sometimes even another; but no mention is ever made of which *is* the best.

We do not think it incumbent upon us to enter into a detailed critical examination of the work: if we did, we should find no difficulty whatever in producing examples to shew that Mr. Cocks sometimes mentions well-known facts in such loose and indefinite language as to lead the reader to no positive conclusion; and that at others he describes operations which are now nearly exploded, omitting any mention of the really "best recorded method," but selecting for the student that which is but rarely practised. Proofs of the carelessness with which the work has been sent from the press are by no means rare. For example, at page 233, we are referred in the text to *Plate 8th* as illustrative of hernia, but Plate 8th illustrates other subjects. Again, at page 306, the reader is referred to Plate 11th for the "surgical means of suppressing hemorrhage," but this plate depicts the appearance of hernia. Mr. Cocks seems to have an insuperable objection, too, to the ordinary mode of spelling the names of the different authorities he quotes: thus, we have "La Dran," "Baron Heurtelop," "Caviale," &c. It may appear hypercritical to mention these mistakes, and we certainly should not have done so if the work, in general, had possessed any redeeming merits. It has often been said, and truly too, that a medical practitioner may fail completely in a court of justice, in giving clear and satisfactory evidence upon a subject with which he is well acquainted, and upon which he would *act* skilfully and efficiently. The same observation applies to written statements, which may be loosely worded or injudiciously compiled, and yet the writer may be a very good and safe practitioner: and this may very probably be the case with Mr. Cocks.

